# Supplementary material for: Steam caps in geothermal reservoirs can be monitored using seismic noise interferometry
Source: Commun Earth Environ. 2023 Dec 2;4(1):453. doi: 10.1038/s43247-023-01122-8 (PMC11041760; doi:10.1038/s43247-023-01122-8)
Supplement: Supplementary file 2 — Supplementary Information [file 43247_2023_1122_MOESM2_ESM.pdf]

Supplementary information

Steam caps in geothermal reservoirs can be monitored using seismic noise interferometry

Pilar Sánchez-Pastor[1,2]*, Sin-Mei Wu[1,3], Ketil Hokstad[4], Bjarni Kristjánsson[5], Vincent Drouin[6], Cécile Ducrocq[7], Gunnar Gunnarsson[5], Antonio Rinaldi[1], Stefan Wiemer[1] and Anne Obermann[1]

[1]Swiss Seismological Service (SED), ETH Zürich, Switzerland

[2]Geosciences Barcelona (GEO3BCN), CSIC, Lluís Solé i Sabarís s/n, Barcelona, Spain

[3]Earth and Environmental Sciences Area, Lawrence Berkeley National Laboratory, Berkeley, CA, USA

[4]Equinor Research Centre, Arkitekt Ebbells vei 10, N-7053 Trondheim, Norway

[5]OR - Reykjavik Energy, Bæjarhálsi 1, 110 Reykjavík, Iceland

[6]Icelandic Meteorological Office, Reykjavík 101, Iceland

[7]Nordic Volcanological Center, Institute of Earth Sciences, University of Iceland, Reykjavík 101, Iceland

(*) psanchezsp@gmail.com

Supplementary figures

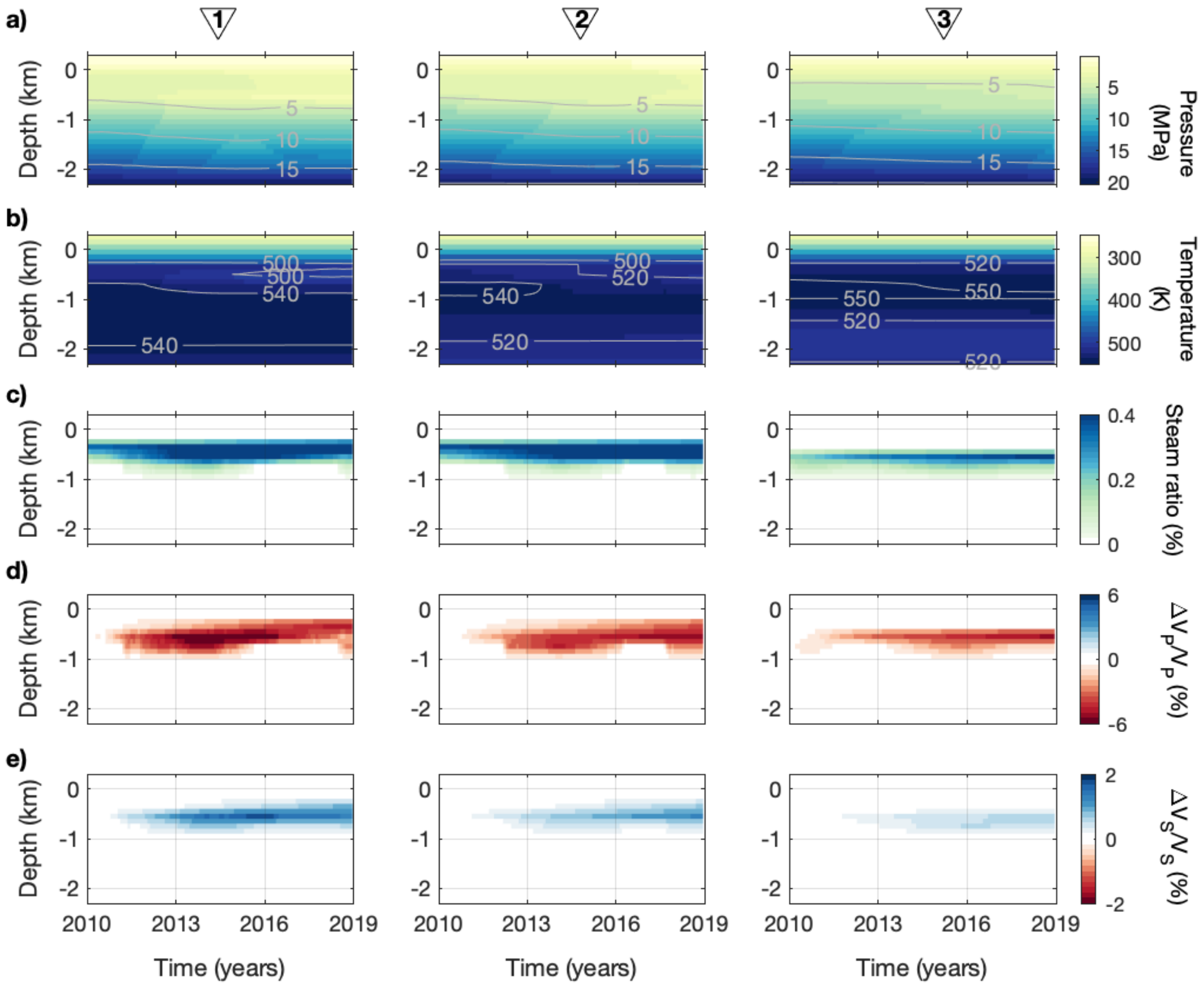

Supplementary Fig. 1 | Thermodynamic and geomechanical models. (a) Decadal pressure evolution obtained from the iTOUGH2 hydrogeological model[1] at the three locations highlighted in Fig. 1b. (b) Same for the temperature (c) Same for the steam ratio (d) Modelled $\Delta V_P/V_P$ and (e) $\Delta V_S/V_S$ using the rock physics model (see Methods subsection Rock physics model).

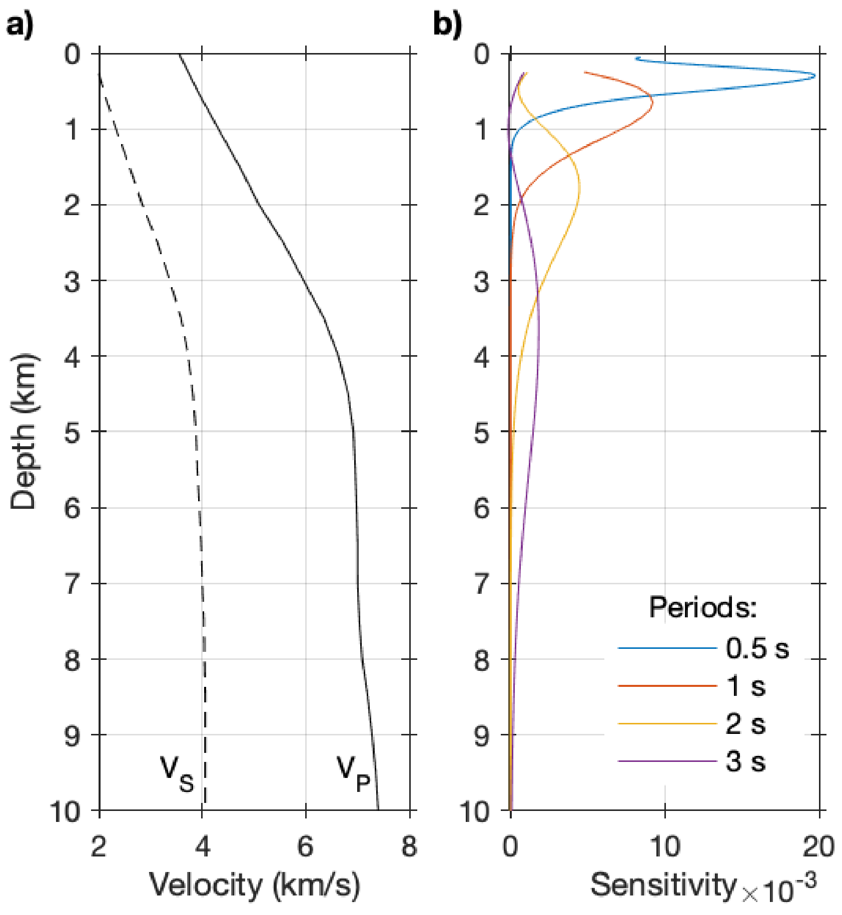

Supplementary Fig. 2 | Depth sensitivity of the $\Delta v/v$ observations. (a) 1D seismic velocity models in the Hengill area[2]. (b) Corresponding 1D depth sensitivity kernels for the fundamental mode of Rayleigh waves[3].

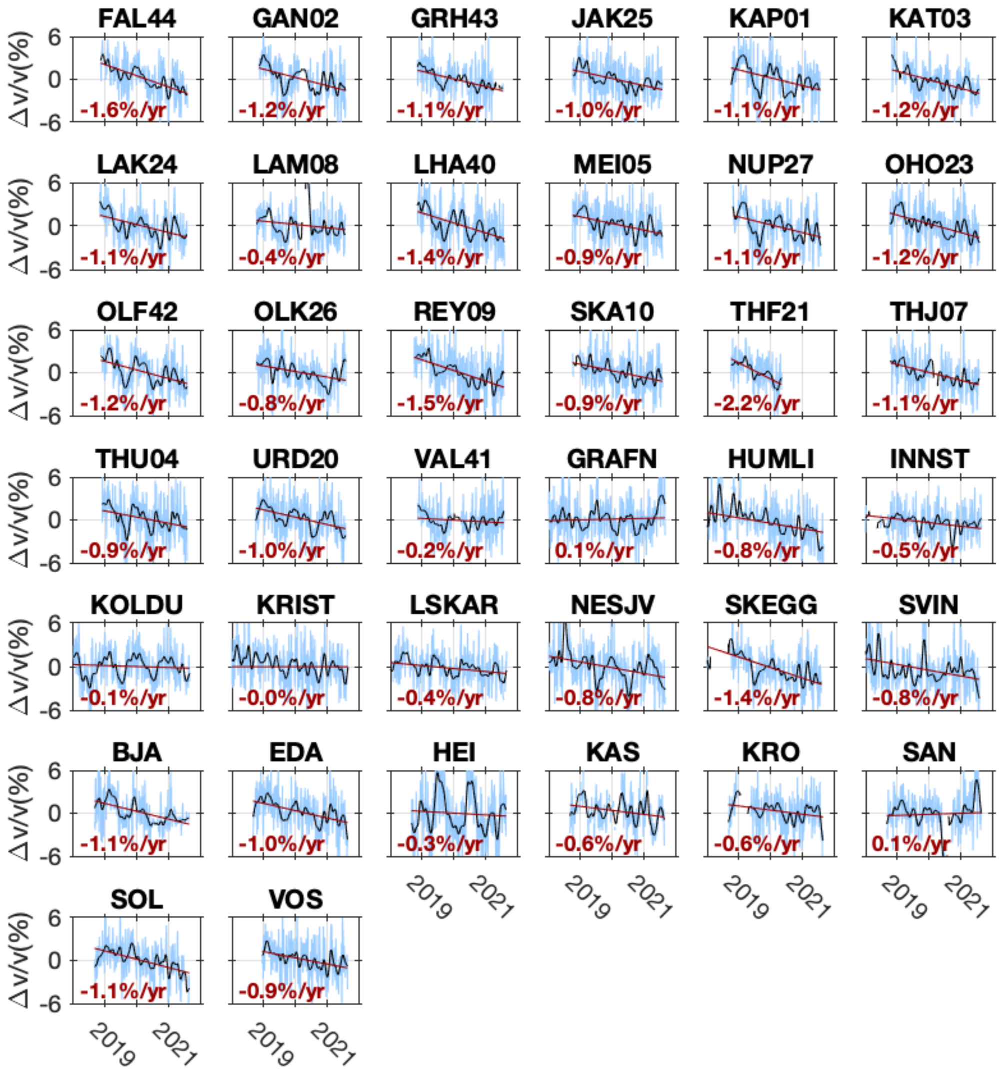

Supplementary Fig. 3 | Overall observed $\Delta v/v$ time series. The estimated $\Delta v/v$ are represented in light blue, the smoothed results employing a Gaussian-weighted moving window of 1-month length are depicted in black. The linear trend and the corresponding annual rate are represented in dark red.

Supplementary references

1. G. Gunnarsson, A. Arnaldsson and A. Oddsdóttir, "Model Simulations of the Hengill Area, Southwestern Iceland," Transport in Porous Media, vol. 90, pp. 3-22, 2011.

2. A. Obermann, S.-M. Wu, T. Ágústsdóttir, A. Duran, T. Diehl, P. Sánchez-Pastor, S. Kristjansdóttir, V. Hjörleifsdóttir, S. Wiemer and G. P. Hersir, "Seismicity and 3-D body-wave velocity models across the Hengill geothermal area, SW Iceland," *Frontiers in Earth Science,* vol. 10, 2022.

3. R. Herrmann, "Computer Programs in Seismology: An Evolving Tool for Instruction and Research," *Seismological Research Letters,* vol. 84, pp. 1081-1088, 24 October 2013.
